# The Genetic Basis of Quality of Life in Healthy Swedish Women: A Candidate Gene Approach

**DOI:** 10.1371/journal.pone.0118292

**Published:** 2015-02-12

**Authors:** Dounya Schoormans, Jingmei Li, Hatef Darabi, Yvonne Brandberg, Mirjam A. G. Sprangers, Mikael Eriksson, Koos H. Zwinderman, Per Hall

**Affiliations:** 1 Department of Medical Epidemiology and Biostatistics, Karolinska Institutet, Stockholm, Sweden; 2 Department of Medical Psychology, Academic Medical Center, Amsterdam, The Netherlands; 3 Department of Medical and Clinical Psychology, Tilburg University, Tilburg, The Netherlands; 4 Human Genetics, Genome Institute of Singapore, Singapore, Singapore; 5 Department of Oncology-Pathology, Karolinska Institutet, Stockholm, Sweden; 6 Department of Clinical Epidemiology and Biostatistics, Academic Medical Center, Amsterdam, The Netherlands; Harvard Medical School, UNITED STATES

## Abstract

**Background:**

Quality of life (QoL) is an increasingly important parameter in clinical practice as it predicts mortality and poor health outcomes. It is hypothesized that one may have a genetic predisposition for QoL. We therefore related 139 candidate genes, selected through a literature search, to QoL in healthy females.

**Methods:**

In 5,142 healthy females, background characteristics (i.e. demographic, clinical, lifestyle, and psychological factors) were assessed. QoL was measured by the EORTC QLQ-C30, which consists of 15 domains. For all women genotype information was available. For each candidate gene, single nucleotide polymorphisms (SNPs) were identified based on their functional (n = 2,663) and physical annotation (n = 10,649). SNPs were related to each QoL-domain, while controlling for background characteristics and population stratification. Finally, gene-based analyses were performed relating the combined effect of 10,649 SNPs (selected based on physical annotation) for each gene, to QoL using the statistical software package VEGAS.

**Results:**

Overall, we found no relation between genetic variations (SNPs and genes) and 14 out of 15 QoL-domains. The strongest association was found between cognitive functioning and the top SNP rs1468951 (p = 1.21E-05) in the GSTZ1 gene. Furthermore, results of the gene-based test showed that the combined effect of 11 SNPs within the GSTZ1 gene is significantly associated with cognitive functioning (p = 2.60E-05).

**Conclusion:**

If validated, the involvement of GSTZ1 in cognitive functioning underscores its heritability which is likely the result of differences in the dopamine pathway, as GSTZ1 contributes to the equilibrium between dopamine and its neurotoxic metabolites via the glutathione redox cycle.

## Introduction

During the last decades quality of life (QoL) is frequently measured as a subjective rating in research and clinical practice. It is a multifactorial concept that consists of a person’s perception of physical, psychological, and social functioning that is often subdivided into several domains (e.g. physical functioning, emotional functioning, cognitive functioning, social functioning, fatigue, and pain).[1] QoL is an increasingly important parameter in both research and clinical practice as it is predictive of mortality and poor health outcomes, such as morbidity, self-management, and health care.[2–4]

QoL is influenced by demographic characteristics (e.g. age, sex, and race), lifestyle factors (e.g. diet and physical activity) and psychological factors, such as mood states and stress.[5–8] A large proportion of variation between individuals remains unexplained. It is therefore suggested that individual genetic predisposition contributes to the perception of QoL.[9] There is increasing evidence for genetic determinants of depression, well-being, pain, and fatigue.[10–14] In addition, family and twin-studies indicate that the heritability for subjective well-being, depression, and anxiety ranges from thirty to as much as fifty percent.[15–19] Moreover, there is ample evidence that the hypothalamic-pituitary-adrenal axis, immune, neuroendocrine, and cardiovascular system are associated with various QoL-domains.[20]

In 2009 an international and interdisciplinary Consortium for Genetics and Quality of Life Research (GeneQol) was initiated.[9] Its main objective is to identify and investigate potential biological mechanisms, genes and genetic variants involved in QoL. The first studies relating genes to QoL have shown that various single nucleotide polymorphisms (SNPs) in cytokine genes and the glutathione metabolic pathway are related to QoL in different patient groups.[21–23] The previous studies are valuable with little generalizability, as they all include only patient samples. The current study is conducted in a sample of healthy women, which increases the generalizability, as the relation between genetics and QoL is examined without the confounding role of diseases. Recently, the GeneQol consortium has provided an overview of biological markers involved in overall QoL and related domains, such as fatigue, pain, negative and positive functioning.[24] They have identified several candidate genes based on an extensive literature search.[24] We aim to perform an empirical study relating these candidate genes to QoL in a healthy female sample.

The specific objectives are (1) to relate SNPs for each of the listed candidate genes to QoL; and (2) to relate the combined effect of SNPs within each gene to QoL.

## Methods

### Study population and procedure

This study utilized data from the Karolinska Mammography Project for Risk Prediction of Breast Cancer (KARMA) study, see www.karmastudy.org for a detailed description. Data used in this manuscript is available upon request through the KARMA Research Platform which can be found at www.karmastudy.org. In short, KARMA collects data and bio-samples each time a participating woman comes for mammography screening or a clinical mammography at one of four Swedish participating hospitals. In Sweden, the national screening program invites all women at 18 month intervals for those 40–55 years, and for those older than 56–74 years at 24 months. Every woman completes a comprehensive online survey. This survey entails more than 250 questions addressing breast cancer related issues such as reproductive history, cancer treatment, and family history of cancer; lifestyle factors (e.g. alcohol and tobacco use); previous medical conditions other than breast cancer; medication use; and QoL. Blood is donated at each visit and processed at the Karolinska biobank. Every six months data from several registries are linked to the KARMA data: the information network for cancer treatment which entails clinical information on breast cancer patients; the Swedish Cancer; Cause-of-Death; Prescription; and In- and Out-patient registers. The KARMA study was approved by the Swedish regional ethical board at the Karolinska Institutet and is conducted in accordance with the Declaration of Helskini.[25] All women gave written informed consent.

### Measurements

**Background characteristics**. *Demographic and clinical factors* All women reported age, educational level, use of painkillers (e.g. paracetamol and ibuprofen) and being on hormone replacement therapy (yes/no) during the last year. Participants’ previous or ongoing medical conditions such as, high blood pressure, hyperlipidemia, myocardial infarction, angina, heart failure, stroke, polycystics ovary syndrome (PCOS), pre-eclampsia, depression, diabetes, bulimia, and anorexia were self-reported.

*Life style factors* Body Mass Index (BMI) was calculated based on women’s weight in kilogram divided by their squared height in meters. Current tobacco use (yes/no) was self-reported—if women either smoked cigarettes or used snuff (a typical Swedish tobacco in moist powder form).

*Psychological factors* The level of stress experienced during the last five years was assessed by one item *“Please state how stressed you have been feeling in the past five years”*. Answers could be given on a 4 point Likert-scale ranging from *‘never stressed’* to *‘always stressed’*. All participants were asked whether they have experienced any of the following life stressors during the last five years: a close relative who died; own divorce or separation; a close friend who died; serious disease or injury; became unemployed; other very stressing event. Finally, the average number of hours of sleep per night was assessed.

**Quality of life**. QoL was measured with the European Organization for Research and Treatment of Cancer Quality of Life questionnaire Core 30 (EORTC QLQ-C30), a cancer specific QoL-questionnaire.[26] It includes global health status, five functional scales (physical; role; emotional; cognitive; and social), three symptom scales (fatigue; nausea or vomiting; and pain), and six single items (dyspnea; insomnia; appetite loss; constipation; diarrhea; and financial difficulties). These scales and items are linearly transformed from 0 to 100. High scores on the global health status/QoL scale indicate a high level of QoL, and high scores on the functional scales indicate a high level of functioning. Conversely, high scores on the symptom scales/items indicate high levels of health problems. The EORTC QLQ-C30 has been validated and is considered to have good psychometric properties.[26]

**Genotyping**. Some of the KARMA participants were genotyped as part of the iCOGS project (www.nature.com/iCOGS) using the iCOGS array. This array was specifically designed to evaluate genetic variants associated with the risk of breast, ovarian and prostate cancer.[27,28] It comprises ~200,000 SNPs, which were selected in samples from large case-control studies in disease-based consortia. A genome-wide imputation of SNPs using the IMPUTE (V.2.0) software, based on the 1000 Genomes Project [(1KGP) Mar 2012 release (updated Apr 19, 2012)], was performed.[29] For the KARMA dataset, the genotypes of 4,310,392 SNPs were successfully called and passed quality control filter (INFO score from IMPUTE > = 0.8 and minor allele frequency > = 0.01). The imputed iCOGS chip has a 60% coverage of what the Illumnia HumanHap550 chip would cover.

*Selection of single nucleotide polymorphisms* The list of candidate genes derived by the GeneQol consortium is continuously updated based on current literature.[24] At the start of this study, the list entailed 139 candidate genes, which were all related to at least one QoL-domain *(S1 Table: The list of 139 candidate genes, which are all related to at least one QoL-domain)*. SNPs for each candidate gene were selected based on both functional and physical annotation (build 37).[30] For the functional annotation, SNPs were selected according to their effects on expression levels, i.e. whether they are expressions of quantitative trait loci (eQTLs) for that gene. Based on the functional annotation 2,663 SNPs were selected for the 139 candidate genes. For the physical-based annotation, a 20 kb window was used where SNPs were categorized based on both their position and linkage disequilibrium (LD) pattern. For the 139 candidate genes 10,649 SNPs were selected based on their physical annotation.

### Statistical analyses

*Background characteristics and quality of life* QoL scores of the selected KARMA women were compared to a Swedish reference population.[31] The QoL scores on the 15 domains were therefore transformed to standard scores based on the scores of an age-matched Swedish reference population. To compare, standard scores were calculated by dividing the difference between the mean scores of the KARMA women and the scores of the age-matched reference population, by the standard deviations of the reference population. The value of the standard scores can be interpreted according to Cohen’s effect size (d), where a score of <0.2 indicates a small, 0.5–0.8 a moderate and >0.8 a large difference. Analyses were performed in SPSS 16.0.

*Relating single nucleotide polymorphisms to quality of life* Initially, possible covariables for the relation between SNPs and QoL were identified. To do so, all background characteristics (listed in Table 1) were related to each of the QoL-domains separately by means of regression analyses. Background characteristics that were associated with QoL (p<0.10) were included as covariables in the subsequent analyses. To control for population stratification [32], principal components analysis (PCA) was performed by EIGENSTRAT V.4.2 (1,2). We visually inspected PCA plots for outliers in terms of ancestry from CEU (northern and western Europe) clusters. Five principal components were retained after inspection of a Scree plot, and included as covariables in subsequent analyses. For the main analyses, regression analyses were used to study the association between SNPs and QoL, while controlling for covariables and the five principal components. Analyses were performed for SNPs selected on functional and physical annotation separately and run in the statistical program PLINK.[33] Bonferonni corrected p-value was set at 3.76E-06 (0.05 divided by (2,663+10,649 SNPs)). For 10 of the 15 QoL-domains the distribution of scores was non-normal. Scores on the cognitive functioning scale were transformed using square root transformation [√(101-raw score)]. On the remaining nine domains a large percentage of women (range from 66.6% to 92.0%) reported the maximum score on the functional scales and the minimum score on the symptom scales/items. These domains were therefore dichotomized; minimum/maximum value versus the remaining answers.

**Table 1:** Background characteristics (demographic, clinical, lifestyle, and psychological factors) (n = 5,142).

	N (%)
**Demographic factors**	
Age in mean years (range)^a^	54.3 (22–88)
Educational level^b^	
Nine year school	497 (9.7)
Gymnasium	1688 (32.9)
University	2525 (49.2)
Other	419 (8.2)
**Clinical factors**	
Being on hormone replacement therapy (yes)	1709 (33.2)
Using painkillers (yes)	4931 (95.9)
Number of medical conditions^c^	
None	2746 (53.4)
One	1509 (29.3)
Two	618 (12.0)
Three	201 (3.9)
Four or more	68 (1.3)
**Lifestyle factors**	
Body mass index (BMI) as mean score (range)^d^	25.2 (17–52)
Using tobacco (yes)	684 (13.3)
**Psychological factors**	
Stress in the last five years^e^	
Never stressed	275 (5.4)
Seldom stressed	1849 (36.4)
Often stressed	2379 (46.9)
Always stressed	571 (11.3)
Number of life stressors	
None	1728 (33.6)
One	2027 (39.4)
Two	955 (18.6)
Three	343 (6.7)
Four or five	89 (1.7)
Hours of sleep^f^	
5 hours or less	207 (4.4)
6 hours	1103 (23.2)
7 hours	2170 (45.7)
8 hours or more	1269 (26.7)

Note: Data is presented as frequencies (percentages) for 5,142 healthy women included in the KARMA study. Age and body mass index are provided in mean (range).

^a^ = information is missing for 1 participant;

^b^ = information is missing for 14 participants;

^c^ = High blood pressure and depression are the most common conditions;

^d^ = for 17 participants information was unavailable;

^e^ = for 68 participants no information was available;

^f^ = information is missing for 393 participants.

*Gene-Based analyses* Gene-based analyses were performed relating the combined effect of 10,649 SNPs (that were selected based on physical location) for each gene to QoL. Analyses were performed for each of the QoL-domains separately, using the Versatile Gene-based Association Study (VEGAS) software.[34] This software package applies a test by using simulations from the multivariate normal distribution by incorporating information on a set of SNPs within a gene while accounting for LD between SNPs. VEGAS uses HapMap populations to estimate patterns of LD for each gene.[34] Statistical significance is assessed adaptively. In the first step, 1000 simulations are run. If the empirical p-value is <0.01, another 10,000 simulations are performed. If, the empirical p-value is <0.001, another 1,000,000 simulations are performed. If an empirical p-value of 0 is reached, no more simulations will be performed.

## Results

### Background characteristics and quality of life

For 5,142 out of 68,334 KARMA women information on both QoL and genotype data was available, and they were therefore included in this study. Women diagnosed with breast cancer before entering KARMA were excluded. Characteristics of the participating women are presented in Table 1. Women’s scores on the QoL domains are presented in Table 2. Overall, the KARMA women reported a good QoL as they appear to function well and report few symptoms. Although the selected KARMA women scored significantly (p<0.01) different on many of the QoL-domains compared to the Swedish reference sample, differences had a small effect size (Cohen’s d<0.3) *(S1 Fig. Comparing quality of life scores of the KARMA women to a Swedish reference sample)*.

**Table 2:** Mean quality of life scores.

	N = 5,142
**EORTC QLQ-C30 DOMAINS**	
Global health/ quality of life	75.8 (22.2)
**Functional scales**	
Physical functioning (highest QoL)	3427 (66.6)
Role functioning (highest QoL)	3825 (74.5)
Emotional functioning	76.1 (22.8)
Cognitive functioning^a^	87.8 (19.2)
Social functioning (highest QoL)	3826 (74.5)
**Symptom scales/items**	
Fatigue	22.4 (20.8)
Nausea and vomiting (highest QoL)	4486 (87.3)
Pain	20.4 (26.5)
Dyspnoea	19.3 (27.0)
Insomnia	25.0 (30.2)
Appetite loss (highest QoL)	4648 (90.4)
Constipation (highest QoL)	4409 (85.8)
Diarrhea (highest QoL)	4556 (88.7)
Financial difficulties (highest QoL)	4725 (92.0)

Note: For global health/quality of life and the functional scales a higher score indicates a better quality of life, whereas for the symptom scales/items a lower score indicates a better quality of life. For the continuous variables (i.e. global health/quality of life; emotional functioning; cognitive functioning; fatigue; pain; dyspnoea; and insomnia) mean scores (SD) are presented. For the dichotomized scales (i.e. physical functioning; role functioning; social functioning; nausea and vomiting; appetite loss; constipation; diarrhea; financial difficulties) frequencies and percentages for the category with the highest quality of life is provided. Please note that for 6, 3, 10, 1, 0, 6, 5, 3, 2, 13, 4, 3, 2, 4, 7 participants respectively information was missing.

^a^ = cognitive functioning was transformed by using square root transformation [√(101-raw score)], ranging from 1–10 with low scores having a better cognitive functioning. The transformed mean score and standard deviation is 2.9 (2.4).

Results of the identification of possible covariables, relating background characteristics to each QoL-domain, are provided in S2 Table *(S2 Table. The association between background characteristics and quality of life using Wald Chi Square test-statistic)*. As expected, age was positively related to mental QoL (e.g. emotional functioning) and negatively to physical QoL (e.g. physical functioning). Overall, the number of medical conditions, stress during the last five years, and the number of life stressors showed the strongest negative association, whereas the number of hours of sleep had the strongest positive relation with the QoL domains.

### Single nucleotide polymorphisms and genes related to quality of life

**Fig 1:**
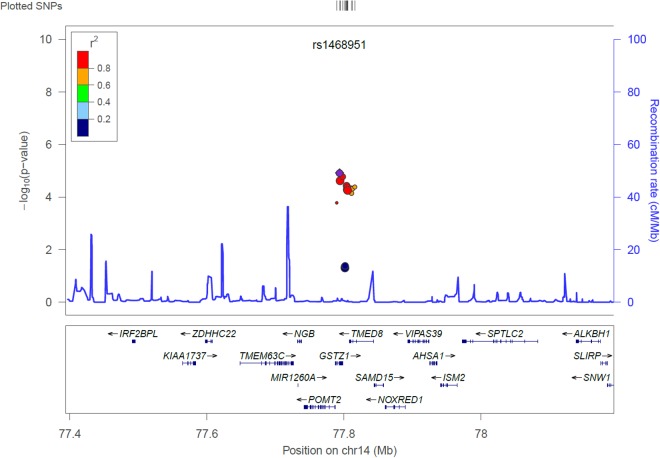
Locus-specific association map generated from genotyped SNPs in the chromosome 14, centered at rs1468951 for cognitive functioning. Note: Vertical axis is the—log10 of the p-value, the horizontal axis is the chromosomal position. Each dot represents a SNP tested for association with cognitive functioning. Linkage disequilibrium (LD) between the most significant SNP, listed at the top of the plot, and the other SNPs in the plot is shown by the r^2^ legend. Locus zoom software was used to prepare this figure.[36]

Results of the association study relating the SNPs selected by functional and physical annotation to QoL, while controlling for possible covariables *(S2 Table. The association between background characteristics and quality of life using Wald Chi Square test-statistic)* are provided in Table 3 and 4 respectively. None of the SNPs selected by functional annotation were significantly related to QoL (Table 3). For SNPs selected based on their physical annotation, there was no statistically significant relation between SNPs and QoL-domains (Table 4). The strongest association was found between cognitive functioning and the top SNP rs1468951 (p = 1.21E-05, Bonferonni-corrected p-value = 3.76E-06) in the *GSTZ1* gene (Table 4), independent of background characteristics (i.e. age, using painkillers, number of medical conditions, using hormone replacement therapy, level of stress in the last five years, number of life stressors, and number of hours of sleep) and the five principal components (controlling for population stratification). This top SNP was an imputed marker, which is in high LD with the genotyped SNP rs1046428 (r^2^ = 0.99). A Manhattan plot of the relation between cognitive functioning and SNPs based on their physical annotation was prepared using Haploview and is displayed in *(S2 Fig. Manhattan plot (p-values per chromosome) for the relation between cognitive functioning and the SNPs found based on physical location for the selected candidate genes)*.[35] The locus-specific association map centered at the top SNP rs1468951 showed low p-values for several SNPs on the *GSTZ1* gene, indicating a relation with cognitive functioning (Fig. 1).[36] To examine the stability of the effect estimate, a sensitivity analysis was performed by sequential omission of individual covariables (leave-one-out analysis). Results revealed that the estimate of rs1468951 remained stable (data not shown).

**Table 3:** Relation between quality of life and the single nucleotide polymorphisms selected by functional annotation (n = 2,663).

FUNCTIONAL ANNOTATION								
	*top SNP*	*Chr*	*Position*	*Minor/major*	*MAF*	*Beta (SE)*	*p*	*GENE*
**QUALITY OF LIFE**								
Global health/ QoL	rs1603406	12	87887139	G/A	0.40	-1.31 (0.41)	1.52E-03	GLDC
**Functional scales**								
Physical functioning	rs10750403	11	128477472	C/T	0.45	0.15 (0.05)	1.36E-03	PRKACA
Role functioning	rs12218712	10	24292743	A/T	0.31	0.18 (0.05)	1.18E-03	HLA-DRB1
Emotional functioning	rs12415866	10	44686664	G/A	0.12	2.15 (0.60)	3.34E-04	RHBDF2
Cognitive functioning^a^	rs17159612	7	84725413	C/T	0.24	-0.16 (0.05)	2.69E-03	SLC6A4
Social functioning	rs1380162	4	119970203	A/G	0.33	0.16 (0.06)	4.71E-03	HSN2
**Symptom scales/items**								
Fatigue	rs1603406	12	87887139	G/A	0.40	1.46 (0.42)	5.56E-04	GLDC
Nausea and vomiting	rs1560580	2	137745374	A/G	0.45	-0.26 (0.07)	1.24E-04	RHBDF2
Pain	rs10150965	14	29018461	G/C	0.41	1.82 (0.52)	5.02E-04	WNK1
Dyspnoea	rs1407818	1	192561712	G/A	0.19	-2.21 (0.68)	1.18E-03	MYB
Insomnia	rs1185701	1	156419617	C/G	0.14	-3.24 (0.79)	4.46E-05	LIPG
Apetite loss	rs10883690	10	83488792	G/T	0.29	-0.28 (0.09)	1.34E-03	PRKACA
Constipation	rs1408808	9	12542187	C/G	0.36	0.20 (0.06)	1.43E-03	UMPS/DRD4
Diarrhoea	rs11848780	14	34169150	A/G	0.13	-0.34 (0.11)	1.29E-03	CD19/MIF/GSTP1
Financial difficulties	rs13160478	5	118082740	G/A	0.11	-0.51 (0.16)	1.04E-03	CASP8

Note: For the 139 candidate genes, 2,663 SNPs were selected based on functional annotation. Bonferonni p-value = 3.76E-06 (0.05/2,663+10,649 SNPs). For the continuous variables (i.e. global health/quality of life; emotional functioning; cognitive functioning; fatigue; pain; dyspnoea; and insomnia) linear regressions were performed. For the dichotomized variables (i.e. physical functioning; role functioning; social functioning; nausea and vomiting; appetite loss; constipation; diarrhea; financial difficulties) we used logistic regression analyses. Chr = chromosome; Position = position of the chromosome; Minor/major = minor and major alleles based on forward strand and minor allele frequencies in Europeans; MAF = minor allele frequency over all European controls in iCOGS; Beta = beta value for the minor allele relative to the major allele; SE = standard error; p = p-value.

^a^ = cognitive functioning was transformed by using square root transformation [√(101-raw score)] ranging from 1–10, with low scores having a better cognitive functioning, therefore the direction of the relation is reversed.

**Table 4:** Relation between quality of life and the single nucleotide polymorphisms selected by physical annotation (n = 10,649).

PHYSICAL ANNOTATION								
	*top SNP*	*Chr*	*Position*	*Minor/ major*	*MAF*	*Beta (SE)*	*p*	*GENE*
**QUALITY OF LIFE**								
Global health/ QoL	rs3783547	2	113533339	G/A	0.37	-1.43 (0.42)	6.66E-04	IL1A
**Functional scales**								
Physical functioning	rs16080	7	24350966	T/C	0.08	0.35 (0.10)	3.28E-04	NPY
Role functioning	rs3889728	1	23084881	C/T	0.25	-0.22 (0.06)	1.14E-04	AGT
Emotional functioning	rs2475376	10	96712400	A/G	0.15	2.04 (0.54)	1.63E-04	CYP2C9
Cognitive functioning^a^	rs1468951	14	77793487	A/C	0.18	0.25 (0.06)	1.21E-05	GSTZ1
Social functioning	rs57758950	8	105453992	T/C	0.13	-0.32 (0.07)	1.63E-05	DPYS
**Symptom scales/items**								
Fatigue	rs2813555	6	152442582	A/G	0.20	1.76 (0.52)	7.56E-04	ESR1
Nausea and vomiting	rs4950025	1	97717279	C/A	0.05	-0.82 (0.19)	1.95E-05	DPYD
Pain	rs35258421	4	142545105	A/G	0.18	2.29 (0.66)	4.86E-04	IL15
Dyspnoea	rs7648614	3	123328980	T/C	0.08	-3.85 (1.08)	3.59E-04	MYLK
Insomnia	rs4298	17	61557200	C/G	0.05	4.09 (1.23)	8.61E-04	ACE
Apetite loss	rs6062900	20	61980125	G/C	0.12	-0.43 (0.12)	6.03E-04	CHRNA4
Constipation	rs324969	7	34791852	G/A	0.48	-0.20 (0.06)	9.11E-04	NPSR1
Diarrhoea	rs748190	10	131519274	G/A	0.46	0.26 (0.07)	2.12E-04	MGMT
Financial difficulties	rs496338	10	131412605	A/T	0.12	-0.59 (0.15)	8.86E-05	MGMT

Note: For the 139 candidate genes, 10,649 SNPs were selected based on physical annotation (build 37). Bonferonni corrected p-value = 3.76E-06 (0.05/2,663+10,649 SNPs). For the continuous variables (i.e. global health/quality of life; emotional functioning; cognitive functioning; fatigue; pain; dyspnoea; and insomnia) linear regressions were performed. For the dichotomized variables (i.e. physical functioning; role functioning; social functioning; nausea and vomiting; appetite loss; constipation; diarrhea; financial difficulties) we used logistic regression analyses. Chr = chromosome; Position = position of the chromosome; Minor/major = minor and major alleles based on forward strand and minor allele frequencies in Europeans; MAF = minor allele frequency over all European controls in iCOGS; Beta = beta value for the minor allele relative to the major allele; SE = standard error; p = p-value.

^a^ = cognitive functioning was transformed by using square root transformation [√(101-raw score)] ranging from 1–10, with low scores having a better cognitive functioning, therefore the direction of the relation is reversed.

Furthermore, results of the gene-based test VEGAS are provided in Table 5. The *GSTZ1* gene (11 SNPs) was significantly associated with cognitive functioning (p = 2.60E-05). For the other domains, none of the genes reached statistical significance after correction for multiple testing. The genotype specific sample and effect sizes for the 11 *GSTZ1* SNPs are provided in S3 Table *(S3 Table: The sample and effect sizes for the 11 SNPs in the GSTZ1 gene)*.

**Table 5:** Gene-based test for 139 candidate genes using the single nucleotide polymorphisms selected by physical location.

QUALITY OF LIFE	Chr	Gene	nSNPs	Start pos	End pos	p
Global health/ QoL	5	IL12B	57	158674368	158690059	1.20E-02
**Functional scales**						
Physical functioning	7	NPY	5	24290333	24298002	8.38E-04
Role functioning	1	AGT	51	228904891	228916959	5.74E-04
Emotional functioning	5	NR3C1	12	142637688	142795270	6.00E-03
Cognitive functioning	14	GSTZ1	11	76857106	76867693	2.60E-05*
Social functioning	10	MGMT	114	131155455	131455358	4.81E-03
**Symptom scales/items**						
Fatigue	12	NR3C1	12	142637688	142795270	1.31E-02
Nausea and vomiting	12	GNB3	1	6819635	6826818	1.13E-03
Pain	1	PER3	51	7767349	7827824	1.21E-02
Dyspnoea	20	GNAS	7	56848189	56919645	5.53E-03
Insomnia	12	AVPR1A	35	61826482	61832857	6.32E-03
Apetite loss	20	CHRNA4	2	61445108	61463139	1.91E-03
Constipation	3	UMPS	56	125931902	125946730	9.73E-03
Diarrhoea	10	BTRC	58	103103814	103307060	1.75E-03
Financial difficulties	7	NPY	5	24290333	24298002	1.49E-03

Note: * p < Bonferonni corrected p-value of 3.60E-04 (0.05/139 candidate genes). Chr = Chromosome; nSNPs = number of SNPs; Test stat = test statistic; p = p-value.

### Identification of causal variants

To identify the causal variant or variants we used two distinct approaches. First, forward selection regression analyses using the step function in R, adjusted for all covariates and principal components, was utilized. The full model included the genotypes of the 11 SNPs annotated to be +-20kb of *GSTZ1* and retained in the VEGAS gene-based analysis. Due to high LD structure in the region a penalty term of five was used [37], with rs11845842 retained in the analysis. Second, we used the ICSNPathway software [38], to explore the coding variant likely responsible for the relation with cognitive functioning. Results showed one candidate causal SNP (rs1046428) and three candidate causal pathways (‘nitrogen compound metabolic process’, ‘anine metabolic process’, ‘amino acid metabolic process’, ‘carboxylic acid metabolic process’, and ‘oxidoreductase activity’). These results indicate the following hypothesis [rs1046428 (non-synonymous coding)-> *GSTZ1* -> nitrogen compound metabolic process’/‘anine metabolic process’/‘amino acid metabolic process’/‘carboxylic acid metabolic process’/‘oxidoreductase activity’ pathways]. Both procedures identified different SNPs, which are in high LD with each other (r^2^ = 0.90), they are thus probably tagging the same causal variant. Furthermore, rerunning the gene-based analysis by excluding our top SNP rs1468951 and the eight *GSTZ1* SNPs in high LD (r^2^>0.9) showed that *GSTZ1* was no longer significantly related to cognitive functioning (p = 4.15E-02). This indicates the cumulative effects in the LD block surrounding rs1468951 within the *GSTZ1* gene.

## Discussion

Overall, we found no relation between genetic variations and 14 out of 15 QoL-domains investigated in this study. For cognitive functioning variations in the *GSTZ1* gene were statistically significant, independent of background characteristics and population stratification.

There are various plausible reasons for the absence of associations between genetic variations and QoL in this study. It is likely that this is—at least in part—the result of limited variation in QoL, due to our healthy female sample. Second, adoption of a candidate gene approach may have resulted in a too limited selection of genes. Furthermore, genotyping was performed by using the iCOGS chip, which was originally built to identify the genetic risk for breast, ovarian and prostate cancer. Although, after imputation, the iCOGS chip covers 60% of what an Illumnia HumanHap550 chip covers, the dispersion over the entire genome may still be skewed. Third, for complex phenotypes a genetic predisposition may be the result of several genes working in concert or the effect of an entire pathway.

The strongest association (p = 1.21E-05, Bonferonni-corrected p-value = 3.76E-06) was found between cognitive functioning and the top SNP rs1468951 in the *GSTZ1* gene, while controlling for background characteristics and population stratification. The imputed marker is in almost perfect LD (r^2^ = 0.99) with the genotyped SNP rs1046428 the latter of which has been annotated in dbSNP as a non-synonymous missense mutation (M [Met] ⇒ T [Thr]), *(S3 Fig. Predicted chromatin state, sequence conservation across mammals, and effect on regulatory motifs of rs1468951 and variants with r^2^ > = 0.8)*.[39] Mining the ENCODE [40] data via HaploReg[39], the intronic variant rs1468951 was predicted to be in DNase hypersensitivity regions in numerous cell lines; and altering predicted relative affinity of two transcription factors (EBF and FXR) *(S4 Fig. Epigenetic road map for rs1468951 effect on regulatory motifs of rs1468951 and variants with r^2^ > = 0.8)*. Mining the RoadMap [41] data predicts rs1468951 to lie in regions in which modification of histone proteins is suggestive in several different cell types (LIV.A, PFM.3 and PFF.2) *(S4 Fig. Epigenetic road map for rs1468951 effect on regulatory motifs of rs1468951 and variants with r^2^ > = 0.8)*. Results of the association between cognitive functioning and the top SNP (rs1468951) without adjusting for background characteristics showed that the relation was not significant [data not shown]. We opted for the inclusion of the covariables based on both literature and the significant findings in the preliminary analyses.[42–45]

In addition, we found that the combined effect of the 11 SNPs within the *GSTZ1* gene were significantly related to cognitive functioning independent of background characteristics, indicating that the multiple smaller effects of the 11 individual *GSTZ1* SNPs seem to be working in concert. This finding is in line with the general understanding that cognitive functioning (e.g. IQ, memory, and concentration) is heritable, and in concordance with the current knowledge of the *GSTZ1* gene. *GSTZ1* encodes multifunctional enzymes important in detoxification and several drugs by conjugation with glutathione. One of these enzymes is maleylacetoacetate isomerase (MAAI) which is involved in the catabolism of phenylalanine and tyrosine.[23,46] Defects in the tyrosine enzyme may lead to severe metabolic disorders including tyrosinaemia which leads to mental retardation and cognitive problems.[47] In experimental studies the administration of tyrosine to individuals under stress leads to improved cognitive functioning, including memory tasks.[48] The physiological basis of this beneficial effect of tyrosine is attributed to its role as precursor for the synthesis of dopamine, which is a major neurotransmitter widely distributed within the brain.[48,49] It is well-known that dopaminergic neurotransmission in the prefrontal cortex contributes to individual differences following a non-linear relation, a so-called reversed U-form.[50] Next to the catabolization of tyrosine into dopamine, *GSTZ1* also contributes to the equilibrium between dopamine and its neurotoxic metabolites via the glutathione redox cycle.[51] Hypothesized is that dopamine and its metabolites have cytotoxic actions on neurons, thereby negatively impacting cognitive functioning, contributing to the U-shaped relation.[52] In a first study relating *GSTZ1* to cognitive functioning among 64–68 aged 470 Scottish community volunteers, a significant association with SNP-1002 G>A was found. A-carriers showed a significantly lower mean score on cognitive functioning, supporting the hypothesis that dopamine disposal pathways may have a negative impact on cognitive functioning.[52]

### Limitations and strengths

There are some noteworthy limitations to this study. As already mentioned, genotyping was performed by using the iCOGS chip, which was originally designed to identify risk factors for breast, ovarian, and prostate cancer. For the gene based analyses we used the VEGAS approach which utilizes HapMap populations to estimate patterns of LD for each gene. In this study, imputations of SNPs were performed by using the 1000 genome reference panel. HapMap has no complete coverage of the number of SNPs per candidate gene as compared to the 1000 genome reference panel.[53] Second, other factors that were not measured could also impact variation in QoL either directly or via genes. For a detailed description of possible relations between biological factors (including genetic markers), background characteristics, and QoL, see the adapted theoretical model of Wilson and Cleary.[20] In addition it is worth mentioning, that we measured subjective cognitive functioning which was assessed by two self-reported items concerning memory and concentration. This two-item scale can be considered limited, however, it has been found to yield high levels of reliability and validity.[26] Furthermore, there is a risk for reporting false positives given the number of tests; for each QoL-domain we related 13,312 SNPs and 139 genes. Finally, this candidate gene study is based on only one study, therefore further validation in independent datasets would be required to confirm the association between *GSTZ1* and subjective cognitive functioning. Since this is a novel area of research the number of studies collecting both genetic and QoL-information are scarce. To the best of our knowledge, no external data are currently available that combine subjective cognitive functioning as assessed with the EORTC QLQ-C30 and genetic data. Since an increasing number of studies are now embarking on the assessment of QoL and genetic data, validation of these data will be possible in the future.

It is important to note that there is controversy in what constitutes QoL. Although QoL can be described as a uni-dimensional concept, we view it as multifactorial consisting of a person’s perception of several domains such as fatigue, physical, emotional, cognitive, and social functioning. One can hypothesize that the more ‘biological’ domains, such as physical and cognitive functioning may have a stronger genetic basis, than for example social functioning. Nevertheless, a recent review reported heritability for social functioning.[54] In our study, there was no significant relation between social functioning and genetic markers. Various possible reasons for this lack of association are provided at the beginning of the discussion. Contrary to our findings, another study examining the relation between genetics and cognitive functioning found a significant association with rs1046428.[55] The most likely explanation for this discrepancy is the difference in measuring cognitive functioning. Where we measured cognitive functioning by self-report examining perceived memory function and concentration, Harris et al. used tasks to examine general mental ability, non-verbal reasoning, verbal fluency and logical memory.[55] These two studies thus examine distinct, albeit related concepts, thereby impeding their comparison.

We would also like to stress the strengths. This is the first study relating QoL to genes in a large sample of healthy females, while statistically controlling for background characteristics including self-reported chronic diseases thereby minimizing the impact of medical conditions. Moreover, the used iCOGS chip has a fairly comprehensive genetic coverage. Furthermore, the included sample of healthy women is representative for the general Swedish population in terms of QoL, increasing generalizability of the results.

### Conclusion and future directions

In conclusion, the involvement of *GSTZ1* in cognitive functioning underscores its heritability which is likely the result of differences in the dopamine pathway. Findings support the hypothesis that dopamine can have negative effects via the neurotoxic by-products.[51] The obvious next step is to replicate the association between cognitive functioning and variations in the *GSTZ1* gene to ensure it is not a chance finding. Although needed, validation is challenging as cognitive functioning is measured in varying ways. In this study measurement entailed two questions tapping into memory and concentration as specific aspects of cognitive functioning.

For future research relating various QoL-domains to genetic markers, a candidate gene approach may be too limited, possibly missing valuable associated genes. Therefore we also opt for a whole genome approach in future studies. Additional research is needed as findings will be valuable in clinical settings. Identified genes can be used as indicators for those who are susceptible to impairments in their QoL. This is particularly useful information for individuals who are experiencing high levels of stress, such as when being diagnosed with or treated for a life-threatening disease. Clinicians may be guided by this information, opting for treatments with the smallest negative impact on QoL and providing additional support to those patients who need it.

## Supporting Information

S1 Table The list of 139 candidate genes, which are all related to at least one QoL-domain. (XLS)

S2 Table The association between background characteristics and quality of life using Wald Chi Square test-statistic. (DOCX)

S3 Table The sample and effect sizes for the 11 SNPs in the GSTZ1 gene. (DOCX)

S1 Fig Comparing quality of life scores of the KARMA women to a Swedish reference sample. [31]. Note: QL = global health/quality of life; PF = physical functioning; RF = role functioning; CF = cognitive functioning; EF = emotional functioning; SF = social functioning; PA = pain; FA = fatigue; NV = nausea and vomiting; SL = insomnia; DY = dyspnoea; AP = appetite loss; CO = constipation; DI = diarrhea; FI = financial difficulties *p<0.01. For example, KARMA women reported better physical functioning, yet more sleeping problems than the Swedish reference sample.[31]
(TIF)

S2 Fig Manhattan plot (p-values per chromosome) for the relation between cognitive functioning and the SNPs found based on physical location for the selected candidate genes. Note: The Bonferonni corrected value is—log10(3.76E-06) = 5.42.This Manhattan plot was prepared using Haploview.[35]
(TIF)

S3 Fig Predicted chromatin state, sequence conservation across mammals, and effect on regulatory motifs of rs1468951 and variants with r^2^ > = 0.8. Note: This figure is a print shot of the haploreg database, see http://www.broadinstitute.org/mammals/haploreg/haploreg.php.[39]
(TIF)

S4 Fig Epigenetic road map for rs1468951 effect on regulatory motifs of rs1468951 and variants with r^2^ > = 0.8. Note: This figure is a print shot of the haploreg database, see http://www.broadinstitute.org/mammals/haploreg/haploreg.php.[39]
(TIF)
