# Supplementary material for: The Genetic Basis of Quality of Life in Healthy Swedish Women: A Candidate Gene Approach
Source: PLoS One. 2015 Feb 12;10(2):e0118292. doi: 10.1371/journal.pone.0118292 (PMC4326277; doi:10.1371/journal.pone.0118292)
Supplement: S3 Table — (DOCX) [file pone.0118292.s003.docx]

| **SNP effect allele** | **0 (n)** | **1 (n)** | **2 (n)** | **Missing (n)** | **Beta heterozygote (SE)** | **Beta homozygote (SE)** | **Beta per-allele (SE)** | **p** |
| --- | --- | --- | --- | --- | --- | --- | --- | --- |
| rs1468951_A | 3428 | 1509 | 155 | 50 | 0.19 (0.07) | 0.42 (0.18) | 0.25 (0.06) | 1.21E-05 |
| rs1046428_T | 3431 | 1546 | 165 | 0 | 0.18 (0.07) | 0.40 (0.18) | 0.24 (0.06) | 2.37E-06 |
| rs1017186_G | 3380 | 1512 | 148 | 102 | 0.16 (0.07) | 0.53 (0.19) | 0.25 (0.06) | 1.67E-05 |
| rs6637_C | 1864 | 2450 | 828 | 0 | -0.02 (0.07) | -0.19 (0.09) | -0.09 (0.04) | 5.0E-02 |
| rs6636_C | 1864 | 2417 | 793 | 68 | -0.02 (0.07) | -0.19 (0.09) | 0.05 (0.04) | 2.34E-01 |
| rs3759731_G | 3388 | 1547 | 154 | 53 | 0.16 (0.07) | 0.52 (0.18) | 0.34 (0.06) | 3.63E-05 |
| rs2160838_T | 3402 | 1554 | 155 | 31 | 0.15 (0.07) | 0.51 (0.18) | 0.23 (0.06) | 5.16E-05 |
| rs9671248_C | 3405 | 1523 | 151 | 63 | 0.16 (0.07) | 0.50 (0.18) | 0.24 (0.06) | 4.26E-05 |
| rs10132619_G | 3425 | 1562 | 155 | 0 | 0.15 (0.07) | 0.50 (0.18) | 0.23 (0.06) | 5.52E-05 |
| rs17105724_G | 3292 | 1526 | 157 | 167 | 0.16 (0.07) | 0.50 (0.18) | 0.24 (0.06) | 4.69E-05 |
| rs11845842_T | 3291 | 1521 | 155 | 175 | 0.16 (0.07) | 0.47 (0.18) | 0.23 (0.06) | 7.12E-05 |

*Table S3: The sample and effect sizes for the 11 SNPs in the GSTZ1 gene*

Note: n = number; Beta = beta value relative to the effect allele; SE = standard error; p = p-value
