# Supplementary figures and images for: The Genetic Basis of Quality of Life in Healthy Swedish Women: A Candidate Gene Approach
Source: PLoS One. 2015 Feb 12;10(2):e0118292. doi: 10.1371/journal.pone.0118292 (PMC4326277; doi:10.1371/journal.pone.0118292)

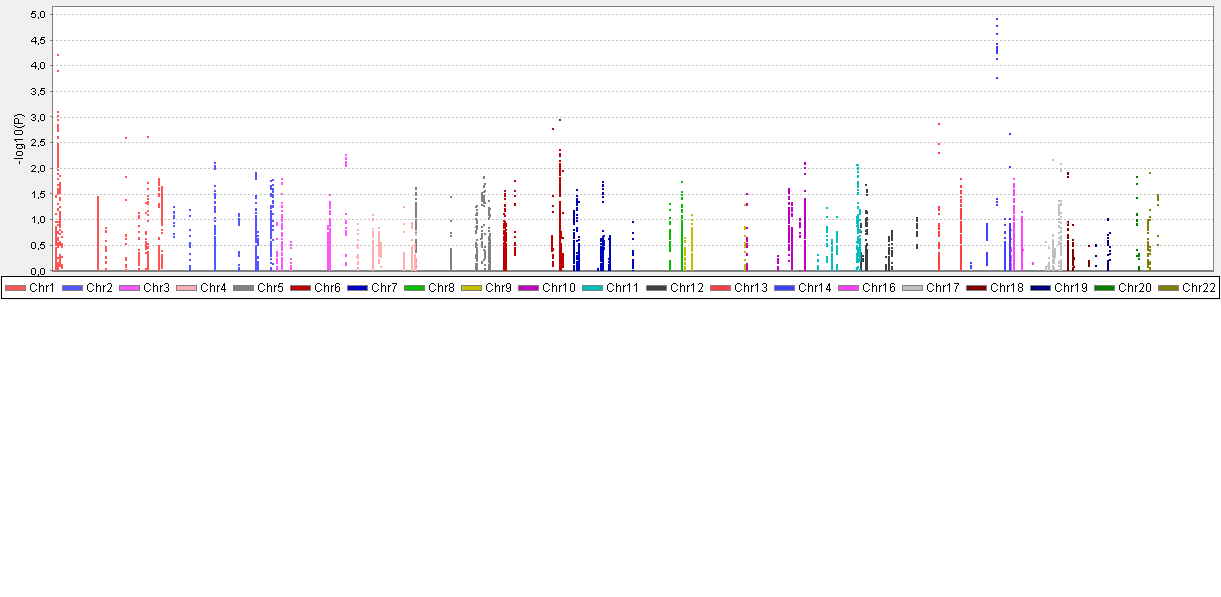

Supplement: S2 Fig — Note: The Bonferonni corrected value is—log10(3.76E-06) = 5.42.This Manhattan plot was prepared using Haploview.[35] (TIF) [file pone.0118292.s005.tif]

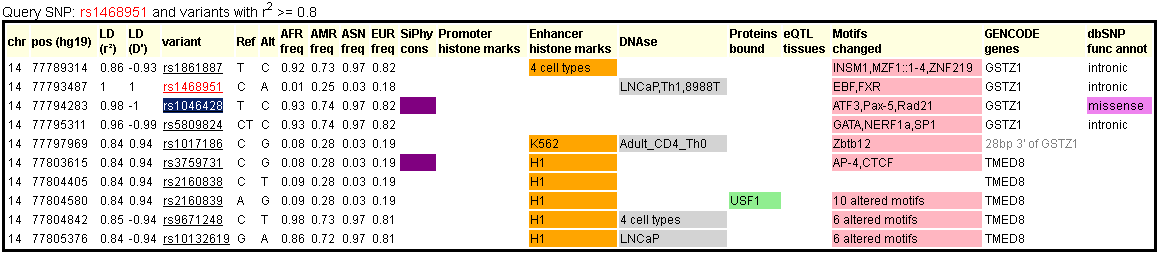

Supplement: S3 Fig — Note: This figure is a print shot of the haploreg database, see http://www.broadinstitute.org/mammals/haploreg/haploreg.php.[39] (TIF) [file pone.0118292.s006.tif]

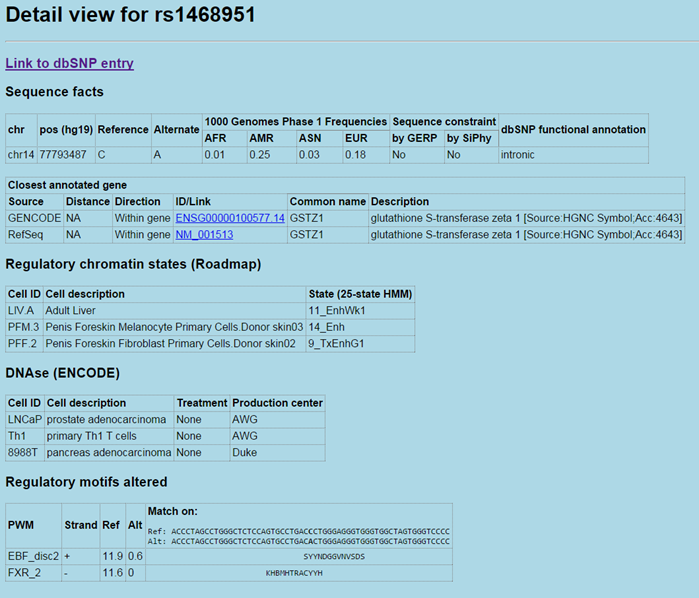

Supplement: S4 Fig — Note: This figure is a print shot of the haploreg database, see http://www.broadinstitute.org/mammals/haploreg/haploreg.php.[39] (TIF) [file pone.0118292.s007.tif]
